# The British Hospitals Association

**Published:** 1912-12-07

**Authors:** Conrad W. Thies

**Affiliations:** Hon. Secretary.


*To the Editor of* The Hospital.

### The British Hospitals Association.

*To the Editor of* The Hospital.

Sir,—With regard to your references last week to the supposed inaction of the British Hospitals Association, will you allow me to inform your readers that a meeting of the Executive Committee had already been summoned previous to the date of your writing? This meeting has since been held, and recommendations prepared for the consideration of the Council, which is to meet shortly. It must be obvious to your readers that, although the hospitals must be prepared with a policy, whether or not an agreement is reached between the Government and the medical profession, yet the problem will be greatly simplified when this question is definitely decided one way or the other. In the expectation of such a decision being arrived at shortly, the Executive Committee purposely delayed summoning the Council. They have, however, employed the interval in collecting information which they trust will assist the Council in their deliberations.—Yours faithfully.

Conrad W. Thies,

Hon. Secretary.

December 3.

[We are glad to receive this letter and to have Mr. Thies' assurance that a policy has been decided upon and will be energetically pursued.—Ed. The Hospital.]

